# A new species of Leucothoid Amphipod, *Anamixis bananarama*, sp. n., from Shallow Coral Reefs in French Polynesia (Crustacea, Amphipoda, Leucothoidae)

**DOI:** 10.3897/zookeys.92.1036

**Published:** 2011-04-28

**Authors:** Thomas James Darwin, Traudl Krapp-Schickel

**Affiliations:** 1 Nova Oceanographic Center, Dania, Florida, USA; 2 Forschungsmuseum A. Koenig, Bonn, Germany

**Keywords:** Amphipod, Leucothoidae, sponge, Coral reefs, French Polynesia

## Abstract

Both leucomorph and anamorph developmental stages of *Anamixis bananarama* **sp. n.**, are illustrated and described from shallow back reef environments of Moorea, French Polynesia. Distinguished by vestigial first gnathopods that persist in post-transformational adult males, this is the second species in the genus to exhibit this unusual character. In other features such as coxae and second gnathopods *Anamixis bananarama* **sp. n.** resembles other Pacific Plate endemics of *Anamixis* known from the region. Specific host association is not documented but suspected to be small calcareous asconoid sponges associated with coral rubble.

## Introduction

Leucothoid amphipods are of interest for their unusual ecology as commensal inhabitants of sessile invertebrates such as sponges, sea squirts, and bivalves. Obligate commensal species have evolved highly characteristic and unusual morphologies and feeding strategies as a consequence of their way of life, including eusocial structure, a condition once thought limited to insects and naked mole rats. (Duffy (1996, 2003) first documented eusocial behavior in marine sponge-inhabiting snapping shrimp. (Thomas (1983, 1997) documented eusociality and communal living in highly derived tropical leucothoids in the genus *Anamixis*. Thiel (1999) reported on nest guarding in *Leucothoe spinicarpa* from Florida, USA. Because of their cryptic lifestyle and need for specialized collecting methods, leucothoid diversity has been vastly underrepresented in museum collections. Specialized *in-situ* underwater collecting techniques pioneered by the first author are beginning to reveal the extent of leucothoid diversity (Thomas 1997, Thomas and Klebba 2006, 2007; White and Thomas 2010). The 22 species in genus *Anamixis* Stebbing, 1897, are primarily tropical – warm temperate in distribution. Their greatest diversity is in the Pacific with 14 species, followed by the Indian Ocean with five species, and the Caribbean Sea and Western Atlantic with four species. Further specialized collecting of host and symbiont will undoubtedly significantly expand the addition of new taxa in the Leucothoidae.

Thomas and Barnard (1983) announced the transformation of males of *Leucothoides pottsi*, originally belonging to Leucothoidae Dana, 1852, into hyperadult males of *Anamixis hanseni* belonging to Anamixidae Stebbing, 1897 [now *Anamixis cavatura* (Thomas, 1997)], but avoided synymymising the two families. In 2000, Lowry and colleagues merged the Anamixidae and Leucothoidae (Lowry et al, 2000). With the addition of both anamorph and leucomorph descriptions in *Anamixis bananarama* sp. n. this proposed familial restructuring now comprises 139 species in six genera: *Anamixis* Stebbing, 1897 (22 spp.); *Nepanamixis* Thomas, 1997 (4 spp.); *Paranamixis* Schellenberg, 1938 (13 spp.); *Leucothoe* Leach, 1814 (96 spp.); *Leucothoella* Schellenberg, 1928 (2 spp.); and *Paraleucothoe* Stebbing, 1899 (2 spp.). A full taxonomic database for the Leucothoidae with hyperlinks to illustrations is available (Thomas, 1999) at: http://www.nova.edu/ocean/jthomas/Current_Leucothoidae_7_09.pdf

In a study of leucothoids from Florida and Belize reefs, the first author and graduate students documented 43 invertebrate host species for Caribbean leucothoids (Thomas and Klebba 2006; 2007). These results combined with the recent addition of Lizard Island (Australia) species of leucothoids underscore the high level of undiscovered leucothoid diversity and illustrate how specialized field collecting can lead to new taxonomic discovery. Despite current taxonomic limitations, the Leucothoidae remain objects of intense interest due to their intriguing ecology, endocommensal lifestyle, and emerging biogeographic patterns.

## Methods

Specimens were collected by snorkeling and SCUBA. Rubble and other shallow algal substrates were isolated *in-situ* and processed by elutriation. In the lab juvenile (leucomorph) and adult males (anamorphs) were separated and photographed using AutoMontage©. Specimens were fixed in 100% ETOH for molecular analysis and in 2% buffered formalin for dissection and illustration. Type material is deposited in the collections of the Florida Museum of Natural History in Gainesville, Florida with the prefix “UF” for museum numbers.

## Taxonomy

### Anamixis bananarama sp. n.

urn:lsid:zoobank.org:act:C57C627F-D2F5-48CD-94C9-5868B6144F96

http://species-id.net/wiki/Anamixis_bananarama

**Figure 1.**
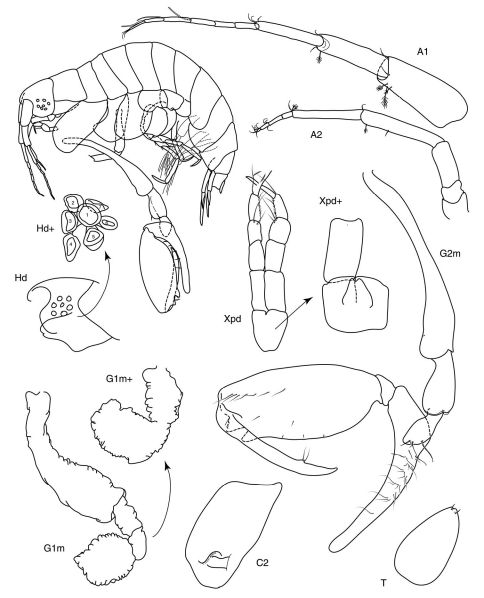
*Anamixis bananarama* sp. n., holotype, anamorph male male ”A”, 2.34 mm.

Figures 1 [Fig F2] 3

#### Type material.

*Holotype*, Anamorph male “A”, 2.34mm, UF 26542, *Paratype*, leucomorph female “B”, 2.10mm; UF 26543; Cook’s Bay, Moorea, French Polynesia, J.D. Thomas collector, 4 December 2009, JDT Moorea 09–4 (South-17.48220:West-149.82530). Wash of backreef rubble, 1–2m. *Additional paratypes*, female leucomorphs (7 specimens), UF 26544, Cook’s Bay, Moorea, French Polynesia, J.D. Thomas collector, 11 December 2009, JDT-Moorea 09–10 (South 17.59205:West-149.835211) coral rubble, coralgal sand, and coral heads, 2–3m.

#### Additional material.

Hans-Georg Mueller collector; 27 February to 6 March 1988, shallow reef, Bora Bora.

#### Diagnosis.

Terminal anamorph males: Eyes with 7 scattered ommatidia; gnathopod 1 greatly reduced, shriveled, persisting in post-transformational stages. Gnathopod 2, basis greatly elongated, narrow; carpus elongate, apically blunt, with reduced setal tufts on medial margin; propodus with sparse mediofacial setal row. Telson elongate for genus, 1.72 times longer than wide.

#### Description.

Head margin broadly rounded, lacking any defining processes; ventral keel, anterior margin rounded, with small midapical indentation; eyes reduced, consisting of 7 scattered ommatidial facets. Antenna 1, ratio of segments 1–3, 43:33:28, peduncle segment 1 and 2 with 3 and 2 plumose setae respectively; flagellum 6-articulate, articles 3–6 with aesthetascs. Antenna 2, flagellum short, 4-articulate. Maxilliped, inner plates fused, apically produced, apical margin with small concave excavation; outer plates lacking inner lobes; palp article 4, 1.36 times length of article 3. Pereonite 1 with small lateral locking ridge.

Gnathopod 1, coxa greatly reduced, apically bifid; remainder of appendage a small bud, articles 2–5 extremely reduced and shriveled. Gnathopod 2, coxa extending deeper than coxa 3–4, distal margin evenly rounded, bearing 10 mediodistal submarginal setules; basis thin, elongate; carpus slightly curved, blunt, reaching 85 percent of propodus, with 6 medial clusters of setae: 2:(2+3):(3+3):(4+1):3:1, distal margin with 5 small submarginal setules; propodus with single row of 7 mediofacial feeding setae, extending 43 percent of propodus length, posterior margin smooth with 3 submarginal setae, anterior margin with 2 prominent apically truncate processes; dactyl straight, inner surface smooth with paired setae on small process near apex, reaching 83 percent on propodus.

Pereopod 3, coxa smaller than 4, rounded ventrally, anterior and posterior margins straight; remainder of pereopods unremarkable. Pereopod 4, coxa slightly larger than 3, posterior margin slightly expanded, remainder of peropod similar to pereopod 3. Pereopods 5–6, coxae bilobed; pereopod 7, coxa entire. Epimera normal for genus. Uropods 1–2, outer rami shortened, approximately 40 percent of inner ramus. Uropod 3, outer ramus 40 percent of inner ramus, outer and inner rami with 1 and 2 marginal spines respectively. Telson 1.70 times longer than wide, with 2 apical setae.

**Leucomorph.** Description of female leucomorph. Head, anterior margin rounded, smooth, eyes consisting of 7 scattered ommatidial facets. Antenna 1, ratio of segments 1–3, 33:26:23; flagellum 5-articulate, articles 4 and 5 with aesthetascs. Antenna 2, flagellum short, 5-articulate. Gnathopod 1, coxa moderately reduced, extending to ventral margin of head, apically bifid; carpus shorter than propodus, with two apical recurved spines, anterior margin bare; propodus, posterior margin finely serrate, bearing a thick recurved apical seta. Gnathopod 2, anterior margin of coxa broadly rounded, ventral margin slightly produced, posteroventral corner with small cusp, posterior margin straight; propodus, palm angle transverse, palmar margin defined by series of concavities and processes, corner of palm defined by distinct cusp; dactyl reaching to end of palm. Telson 1.72 times longer than wide, with 2 apical setae.

**Figure 2. F2:**
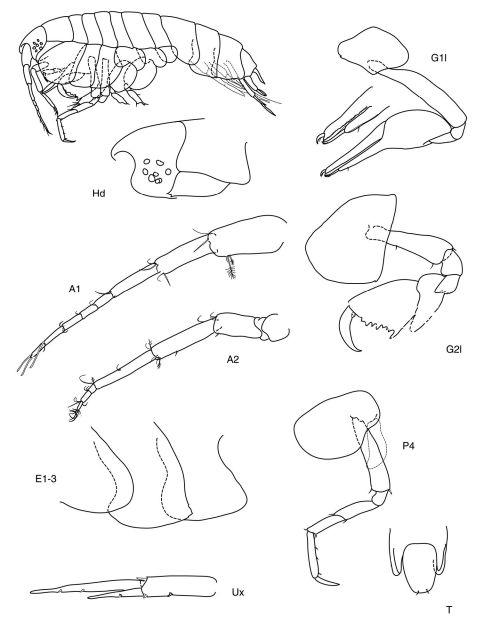
*Anamixis bananarama* sp. n., paratype, leucomorph female “B”, 2.10mm.

**Figure 3.**
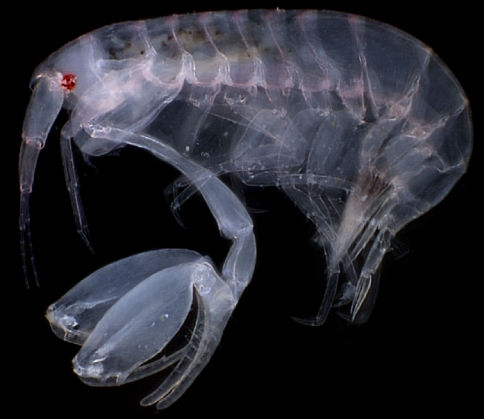
AutoMontage Z-stacked photograph of *Anamixis bananarama* sp. n., holotype, anamorph male male ”A”, 2.34 mm.

#### Etymology.

This species is named for the long recurved carpal lobe on the second gnathopod that resembles the shape of a banana.

#### Relationships.

*Anamixis bananarama* shares it closest affinity with *Anamixis jebbi* from the Madang Lagoon, Papua New Guinea, (Thomas, 1997), with both species having 7 ommatidial facets in both leucomorph and anamorph stages, and a reduced, vestigial first gnathopod in the post transformational anamorph stage. The second gnathopod of *Anamixis bananarama* differs from *Anamixis jebbi* in the elongate basis, the more blunt and less setose carpus, and reduced mediofacial setal row (7 in *Anamixis bananarama*; 14 in *Anamixis jebbi*). *Anamixis bananarama* exhibits an elongated telson typical of ratios found in *Nepanamixis* (Thomas, 1997). Both *Anamixis jebbi* and *Anamixis bananarama* show transitional characters placing them in a clade by themselves with the elongate telson of *Anamixis bananarama* placing it nearer to *Nepanamixis* in this regard. The telson of *Anamixis bananarama* at 1.70 times longer than wide exceeds that of *Anamixis jebbi* at 1.32 and approaches the telson ratios typical of the genus *Nepanamixis* at 1.8–2.0 times longer than wide.

#### Remarks.

Color in life and in freshly collected and preserved material of both leucomorph and anamorph stages are pale translucent pink. There is faint thin reddish banding on posterior thoracic and abdominal segments. Eyes are red. Ovigerous females contain an average of 7–10 yellow eggs in the marsupium.

The vestigial first gnathopods found in *Anamixis bananarama* and *Anamixis jebbi* are persistent morphologies in post transformational anamorphs in both taxa. A number of specimens were examined by the first author to ensure these were not transitional transformational features as reported by Thomas (1997) in *Paranamixis clarkae* from the Seychelles Islands. In *Paranamixis clarkae*, transformational anamorphs exhibit small shrunken vestiges of gnathopod 1 which are lost in subsequent molts. Ren (2006) described *Paranamixis vestigium* from the South China Sea, illustrating similar reduced first gnathopods. In all other aspects *Paranamixis vestigium* resembles *Paranamixis misakiensis* described by Thomas (1997) from Japan and examination of a series of anamorph specimens of *Paranamixis vestigium* is needed to resolve whether these vestigial first gnathopods persist in post-transformational molts.

#### Habitat.

Specific habitat/host undocumented but assumed to be small asconoid calcareous sponges in protected rubble habitats in backreef environments.

#### Distribution.

Moorea and Bora Bora, French Polynesia, Pacific Ocean. 1–3m.

## Supplementary Material

XML Treatment for Anamixis bananarama
